# Relationships between aeroallergen levels and hospital admissions for asthma in the Brussels-Capital Region: a daily time series analysis

**DOI:** 10.1186/s12940-018-0378-x

**Published:** 2018-04-11

**Authors:** Ariane Guilbert, Bianca Cox, Nicolas Bruffaerts, Lucie Hoebeke, Ann Packeu, Marijke Hendrickx, Koen De Cremer, Sandrine Bladt, Olivier Brasseur, An Van Nieuwenhuyse

**Affiliations:** 1 Health and Environment Unit, Scientific Institute of Public Health, Rue Juliette Wytsmanstraat 14, 1050 Brussels, Belgium; 2 Centre for Environmental Sciences, Hasselt University, Campus Diepenbeek Agoralaan Gebouw D, 3590 Diepenbeek, Belgium; 3 Mycology and Aerobiology Unit, Scientific Institute of Public Health, Rue Juliette Wytsmanstraat 14, 1050 Brussels, Belgium; 4 Laboratory and Air Quality Department, Brussels Environment, Avenue du Port 86c-3000, 1000 Brussels, Belgium

**Keywords:** Asthma, Hospitalization, Pollen grain, Fungal spore, Air pollution, Time series, Distributed lag model

## Abstract

**Background:**

Outdoor pollen grain and fungal spore concentrations have been associated with severe asthma exacerbations at the population level. The specific impact of each taxon and the concomitant effect of air pollution on these symptoms have, however, still to be better characterized. This study aimed to investigate the short-term associations between ambient concentrations of various aeroallergens and hospitalizations related to asthma in the Brussels-Capital Region (Belgium), an area recording especially high rates of admissions.

**Methods:**

Based on administrative records of asthma hospitalizations and regular monitoring of 11 tree/herbaceous pollen taxa and 2 fungal spore taxa, daily time series analyses covering the 2008–2013 period were performed. Effects up to 6 days after exposure were captured by combining quasi-Poisson regression with distributed lag models, adjusting for seasonal and long-term trends, day of the week, public holidays, mean temperature and relative humidity. Effect modification by age and air pollution (PM, NO_2_, O_3_) was tested.

**Results:**

A significant increase in asthma hospitalizations was observed for an interquartile range increase in grass (5.9%, 95% CI: 0.0, 12.0), birch (3.2%, 95% CI: 1.1, 5.3) and hornbeam (0.7%, 95% CI: 0.2, 1.3) pollen concentrations. For several taxa including grasses, an age modification effect was notable, the hospitalization risk tending to be higher in individuals younger than 60 years. Air pollutants impacted the relationships too: the risk appeared to be stronger for grass and birch pollen concentrations in case of high PM_10_ and O_3_ concentrations respectively.

**Conclusions:**

These findings suggest that airborne grass, birch and hornbeam pollen are associated with severe asthma exacerbations in the Brussels region. These compounds appear to act in synergy with air pollution and to more specifically affect young and intermediate age groups. Most of these life-threatening events could theoretically be prevented with improved disease diagnosis/management and targeted communication actions.

**Electronic supplementary material:**

The online version of this article (10.1186/s12940-018-0378-x) contains supplementary material, which is available to authorized users.

## Background

Asthma represents a complex and heterogeneous respiratory disorder. It is characterized by a chronic inflammation of the lower airways, leading to variable and recurring respiratory difficulties such as wheezing, chest tightness, coughing, shortness of breath, etc. [1]. It severely impairs patients’ quality of life and requires the use of long-term control medicines as well as quick-relief ones in case of symptoms exacerbations [2]. Despite these treatments, failures in disease control may occur and asthma hospitalizations or even death are still frequent [3].

The prevalence of this disease, and its associated burden on global health care systems, greatly increased over the last decades. Nowadays, around 334 million people are estimated to be affected worldwide, making this disease a major public health concern [3]. Belgium shows among the highest prevalence rates in Europe: 9.83% of the adult population (18 to 45 years) was medically diagnosed for asthma in the early 2000s [4]. The country also records high figures of asthma hospitalizations, ranking fifth in a list of 28 European countries according to the WHO Hospital Morbidity Database [3, 5]. At regional level, the highest rates are observed in the Brussels-Capital Region [unpublished observations].

For several years, research has attempted to identify the triggers contributing to severe asthma exacerbations. A number of environmental factors appear to be involved, especially for the allergic form of the disease. Among outdoor parameters, air pollution and weather conditions have been widely recognized as (in)directly responsible for day-to-day variation in asthma hospitalizations [6–9]. Also, airborne concentrations of some pollen [10–17] and fungal spore [10, 18] taxa have been found to be associated with increases in asthma admissions. However, many of these studies focused on only a limited number of plant/fungal aeroallergen taxa or on grouped taxa, while each individual aeroallergen presents specific allergy potency and can trigger various symptoms in distinct populations. Conclusions on which specific taxa are associated with asthma hospitalizations, as well as reported effect sizes, tend to vary across studies, probably influenced by the local context and the methodology used. Moreover, only few studies have investigated potential effect modification by air pollutants and confounding by respiratory infections in the association between pollen/fungal spore concentrations and asthma hospitalizations.

Green spaces represent a significant part of the Brussels territory (54%, of which 82% are considered as dense vegetation) [19] and the area is characterized by an intense pollen season [20]. Besides, recently published studies highlighted an increasing trend of pollen concentrations for trees with allergy potency [20, 21]. In combination with the high asthma hospitalization rates registered in the Brussels-Capital Region [3, 5], a study on the associations of pollen and fungal spore concentrations with asthma hospitalizations appeared warranted. Ultimately, conclusions should allow the development of more targeted prevention strategies against outdoor aeroallergens.

## Methods

### Aim, design and setting

This study aimed to analyse the short-term relationships of airborne pollen and fungal spore concentrations with hospital admissions for asthma, considering the potential modifying effects of age and air pollution.

To do so, an ecological time series approach was adopted, capitalizing on routinely collected data provided by the federal health services and other public monitoring institutions. This protocol was approved by the Belgian Commission for the Protection of Privacy.

The research focused on the Brussels-Capital Region in Belgium. This urban area includes the country capital and represents the largest agglomeration of the state with 1.0–1.1 million inhabitants spread over 161 km^2^ during the studied time period. This study covered six outdoor aeroallergen seasons, from 2008 to 2013 included.

### Data

The primary health outcome investigated for this study was the daily number of hospital admissions with a main/first diagnosis of asthma (coded 493 according to the International Classification of Disease-9) that were registered in the Brussels-Capital Region between the 1st January 2008 and the 31st December 2013. These figures were provided by the Federal Public Service Health, food chain safety and environment. They were derived from the Belgian Minimal Hospital Summary database: any Belgian general hospital is required to systematically generate a standardized summary of the medical record of every inpatient (with a few exceptions). This record includes information on diagnosis, medical services involved, dates of admission and discharge, patient’s demographic characteristics, etc.

Based on their local presence and their relevance for allergy, 11 pollen taxa were selected a priori for this study: alder (*Alnus* spp.), hazel (*Corylus avellana*), yew (*Taxus* spp.) and Cupressaceae (considered together due to impossible visual differentiation), ash (*Fraxinus excelsior*), hornbeam (*Carpinus betulus*), birch (*Betula* spp.), oak (*Quercus* spp.), plantain (*Plantago* spp.), grasses (Poaceae) and mugwort (*Artemisia* spp.). Two fungal spore taxa belonging to the Ascomycetes group were also considered: *Alternaria* spp. and *Cladosporium* spp. Their mean daily concentrations (grains or spores/m^3^ of air) for the area of interest were supplied by the Mycology and Aerobiology unit of the Belgian Scientific Institute of Public Health. This unit is responsible for the national aerobiological surveillance network [22]. It ensures the continuous monitoring of the outdoor air, from January to September for pollen and to November for fungal spores, by using a Hirst-type volumetric spore sampler (Burkard Manufacturing Co., Rickmansworth, UK) placed on the flat and unobstructed roof of a 16 m high building, located in the centre of the study area.

The role of four air pollutants as confounders or effect modifiers was investigated: PM_2.5_, PM_10_, O_3_ and NO_2_. Their population-weighted average 24-h concentrations (μg/m^3^ of air) for the Brussels-Capital Region were made available by the Belgian Interregional Environment Agency. They were derived from a monitoring network of fixed stations spread over the whole region (10 stations during the study period), augmented by a land-use regression model (RIO-CORINE) [23].

The influence of temperature (°C) and relative humidity (%) parameters was considered. The data were supplied by the Royal Meteorological Institute of Belgium as 24-h averages measured within the study area (station of Uccle, Brussels).

Lastly, potential confounding by influenza episodes and general respiratory infections was taken into account. Data on weekly consultation rates for influenza-like illnesses were obtained from the representative Belgian Sentinel General Practitioner network, coordinated by the Scientific Institute of Public Health [24]. Influenza epidemics were defined as weeks (Monday to Sunday) with an incidence above the threshold of 141 cases per 100,000 inhabitants [24]. The daily number of hospital admissions for general respiratory infections was derived from the Belgian Minimal Hospital Summary database.

### Statistical analyses

The associations between outdoor aeroallergen concentrations and hospitalizations for asthma were investigated using daily time series. Analyses were limited to the months of the year for which the pollen or fungal spore taxa under study were present in the air (i.e. months for which the probability that the concentration is higher than 1% of the maximum concentration is different from zero): alder (January–April), hazel (January–April), yew & Cupressaceae (February–April), ash (February–May), hornbeam (March–May), birch (March–May), oak (April–May), plantain (April–September), grasses (April–September), mugwort (June–September), *Alternaria* (January–November) and *Cladosporium* (January–November). Potential delayed effects of aeroallergens on asthma hospitalizations up to 6 days after the exposure were allowed by combining quasi-Poisson regression with distributed lag models [25]. A distributed lag (non-linear) model (DL(N)M) is defined through a “cross-basis” function, which allows the simultaneous estimation of a (non-linear) exposure-response association and non-linear effects across lags, the latter termed lag-response association. A linear exposure-response function was assumed and the lag structure was modelled with a natural cubic spline with 4 degrees of freedom (df). The knots in the lag space were set at equally spaced values in the log scale of lags to allow more flexible lag effects at shorter delays [25].

To capture the (potentially delayed) effects of meteorological factors on asthma hospitalizations, cross-bases for mean temperature and for mean relative humidity were also included in the model. As for aeroallergens, a maximum lag of 6 days with 4 df was used for the lag-response function. The exposure-response functions were modelled using natural cubic splines with 5 df for temperature and 3 df for humidity, placing knots at equally spaced values of the actual temperature/humidity ranges to allow enough flexibility in the two ends of the distributions. Seasonality and long-term trends were modelled using natural cubic splines with equally spaced knots every 30 days of observation. Models were additionally adjusted for indicator variables for day of the week and public holidays.

In secondary analyses, confounding by air pollution was investigated by adding a cross-basis for each air pollutant in separate models. A maximum lag of 6 days and 4 df were used for the lag-response function and a linear exposure-response function was assumed. Confounding by influenza and by general respiratory infections was also assessed by including a binary variable for influenza epidemics and daily counts of hospitalizations for general respiratory infections in the model respectively. Effect modification by age group (0–14, 15–59 and 60 or more years) and by air pollution (below or above the median and the 85th percentile) were investigated through an interaction between the cross-basis for the aeroallergen and indicator variables for age group and air pollution respectively [26]. Effect modification was formally tested by comparing models with and without the interaction term (Wald test on 4 degrees of freedom). To avoid missing important interactions because of lack of power, the significance level for the interaction term was set at 0.15.

In sensitivity analyses, the robustness of results was assessed with respect to the adjustment for temporal trends (by testing knots every 15 or 60 days of observation instead of every 30 days) and with respect to the specification of the lag structure (by using an unconstrained lag model [27]).

Reported estimates represent the cumulative (lag 0–6 days) percentage change (with 95% confidence intervals [CI]) in asthma hospital admissions for an interquartile range increase in aeroallergen concentration. All analyses were performed with the statistical software R (R Foundation for Statistical Computing, Vienna, Austria) using the “dlnm” package [28].

## Results

Five thousand ninety-four hospitalizations for asthma involving individuals from the Brussels-Capital Region were registered between 2008 and 2013. 35% of asthma hospitalizations were in the age group 0–14 years, 41% in the age group 15–59 years and 24% in the age group 60 years and more. The daily number of admissions ranged between 0 and 11 with an average (± standard deviation) of 2.3 (± 1.7). The average daily number was the highest in September (3.5) and the lowest in July and August (around 1.4). Besides, admissions were the most frequent on Mondays (2.8) and the least frequent on Saturdays (1.9).

The distribution of daily pollen and fungal spore concentrations during their respective seasons is presented in Table 1. Exposure to the selected pollen taxa mainly occurred between January and beginning of September. Daily mean concentrations strongly varied, fluctuating between 0 grains/m^3^ for plantain and 82 grains/m^3^ for birch. Exposure to fungal spores took place from January to November, the highest daily concentrations being registered for *Cladosporium* (daily mean: 2731 spores/m^3^). Daily concentrations of some aeroallergens were correlated. Spearman coefficients ranged from 0.01 between alder and birch to 0.78 between *Alternaria* and *Cladosporium* (see Additional file 1)*.*

**Table 1:** Descriptive statistics on pollen and fungal spore levels, Brussels-Capital Region, 2008–2013

Taxa	Aeroallergen season^a^	Number of days included in the analyses	Daily concentrations within the defined aeroallergen season (grains or spores/m^3^)						
			Mean	S.D.	Min	P25	P50	P75	Max
Alder	January–April	722	16	48	0	0	1	9	598
Hazel	January–April	722	6	18	0	0	1	4	207
Yew Cupressaceae	February–April	536	65	195	0	1	9	50	2595
Ash	February–May	722	16	76	0	0	0	4	1265
Hornbeam	March–May	552	4	23	0	0	0	1	387
Birch	March–May	552	82	230	0	0	3	40	1933
Oak	April–May	366	36	89	0	0	2	22	560
Plantain	April–September	1098	0	1	0	0	0	0	10
Grass	April–September	1098	14	26	0	0	3	12	205
Mugwort	June–September	732	1	2	0	0	0	1	21
*Alternaria*	January–November	2006	57	148	0	0	10	40	1985
*Cladosporium*	January–November	2006	2731	4519	0	250	970	3213	43230

Note: ^a^ Months for which the probability that the concentration is higher than 1% of the maximum concentration is different from zero

Descriptive statistics for meteorological variables and air pollutants are given in Table 2.

**Table 2:** Descriptive statistics on meteorological and air pollution conditions, Brussels-Capital Region, 2008–2013

Variables	Mean	S.D.	Min	P25	P50	P75	P85	Max
Mean temperature (°C)	10.7	6.6	−9.2	6.1	11.0	15.8	17.6	28.1
Relative humidity (%)	77.8	12.2	36	69	80	87	91	99
PM_2.5_ (μg/m^3^)	19.0	12.5	3.9	10.6	15.5	23.4	30.1	96.5
PM_10_ (μg/m^3^)	25.6	14.4	7.7	15.7	21.5	31.4	38.8	118.4
O_3_ (μg/m^3^)	36.0	19.6	1.9	21.1	36.2	49.1	56.4	105.9
NO_2_ (μg/m^3^)	34.3	13.8	8.0	23.8	32.2	42.9	48.8	115.3

Note: Air pollution concentrations expressed as spatial and population weighted means

Asthma hospitalizations were significantly associated with hornbeam, birch and grass pollen concentrations, but not with concentrations of other pollen or fungal spore taxa (Table 3). The change in hospitalizations for an interquartile range increase in pollen concentrations was 0.7% (95% CI: 0.2, 1.3), 3.2% (95% CI: 1.1, 5.3) and 5.9% (95% CI: 0.0, 12.0) for hornbeam, birch and grasses respectively. Although not significant, a relatively large negative estimate for oak was observed ( -5.6%, 95% CI: -11.3, 0.5).

**Table 3:** Cumulative (lag 0–6 days) percentage change (95% confidence interval) in asthma hospitalizations associated with an interquartile range increase in pollen or fungal spore concentration, Brussels-Capital Region, 2008–2013

Taxon	Interquartile range (grains or spores /m^3^)^†^	Percentage change (95% CI)
Alder	9	0.2 (−2.2, 2.7)
Hazel	4	0.5 (−2.5, 3.6)
Yew Cupressaceae	49	−2.5 (−6.9, 2.1)
Ash	4	0.0 (−0.6, 0.5)
Hornbeam	1	0.7 (0.2, 1.3)*
Birch	40	3.2 (1.1, 5.3)*
Oak	22	−5.6 (−11.3, 0.5)
Plantain	1	4.1 (−11.9, 22.9)
Grass	12	5.9 (0.0, 12.0)*
Mugwort	1	1.4 (−4.4, 7.6)
*Alternaria*	40	−0.8 (−3.8, 2.3)
*Cladosporium*	2962.5	1.2 (−6.9, 10.0)

Note: Models adjusted for seasonal and long-term trends, day of the week, public holidays and DLNM cross-bases for mean temperature and relative humidity

^†^Calculated based on the aeroallergen season defined for each taxa (equal to 0 grains/m^3^ for plantain but cumulative percentage change calculated for an increase of 1 grain/m^3^)

* *P* < 0.05

No strong evidence of confounding by air pollution was found: although the inclusion of air pollutants had the tendency to increase effect estimates of aeroallergens, the estimate for grasses slightly decreased and became insignificant after adding the cross-basis for NO_2_ to the model (5.3%, 95% CI: -0.5, 11.5) (see Additional file 1). Results were also robust to the inclusion of influenza epidemics and general respiratory infections in the model.

Significant interactions between aeroallergen concentrations and age group were observed for plantain (*P* = 0.11), grasses (*P* = 0.05), mugwort (*P* < 0.01), *Alternaria* (*P* = 0.10) and *Cladosporium* (*P* = 0.13) (Table 4). The estimate for grasses was only significant and considerably higher for individuals aged between 15 and 59 years old (7.9%, 95% CI: 1.7, 14.4). Although not significant, percentage changes for mugwort, *Alternaria* and *Cladosporium* were close to or higher than 0 in the age groups 0–14 and 15–59 years and firmly negative in the elderlies. A significant negative relationship was detected for oak in the oldest age group too (-7.2%, 95% CI: -13.2, -0.8). Exclusion of the patients aged between 0 and 4 years did not significantly affect the conclusions (see Additional file 1).

**Table 4:** Cumulative (lag 0–6 days) percentage change (95% confidence interval) in asthma hospitalizations associated with an interquartile range increase in pollen or fungal spore concentration, by age group, Brussels-Capital Region, 2008–2013

Taxon	Percentage change (95% CI)			
	0–14 years	15–59 years	60 or more years	*P* interaction
Alder	−1.0 (−3.7, 1.7)	0.0 (−2.6, 2.6)	1.0 (−1.5, 3.7)	0.16
Hazel	−0.1 (−3.3, 3.2)	1.0 (−2.1, 4.2)	0.5 (−2.7, 3.8)	0.60
Yew Cupressaceae	−3.9 (−8.8, 1.4)	−3.0 (−7.9, 2.2)	−1.2 (−6.1, 4.1)	0.35
Ash	−0.1 (−0.6, 0.5)	−0.2 (−0.9, 0.4)	0.1 (−0.5, 0.7)	0.37
Hornbeam	0.7 (0.1, 1.3)*	0.8 (0.2, 1.3)*	0.7 (0.1, 1.3)*	0.73
Birch	3.3 (1.1, 5.5)*	3.3 (1.1, 5.6)*	2.8 (0.6, 5.1)*	0.50
Oak	−5.7 (−11.6, 0.5)	−5.0 (−10.9, 1.3)	−7.2 (−13.2, − 0.8)*	0.23
Plantain	7.4 (−9.9, 28.1)	−2.1 (−19.1, 18.5)	4.7 (−13.4, 26.5)	0.11°
Grass	5.2 (−0.9, 11.5)	7.9 (1.7, 14.4)*	4.1 (−2.1, 10.7)	0.05°
Mugwort	1.3 (−4.8, 7.9)	5.3 (−1.0, 12.1)	−4.2 (−10.8, 2.9)	< 0.01°
*Alternaria*	−0.3 (−3.3, 2.9)	0.2 (−2.9, 3.4)	−3.2 (−6.5, 0.2)	0.10°
*Cladosporium*	2.3 (−6.1, 11.3)	2.8 (−5.6, 12.0)	−2.7 (−11.0, 6.3)	0.13°

Note: Models adjusted for seasonal and long-term trends, day of the week, public holidays and DLNM cross-bases for mean temperature and relative humidity

* *P* < 0.05

° *P* interaction < 0.15 (significant effect modification by age group)

Potential effect modification by air pollutants was tested in separate analyses (Table 5). A significant effect modification by PM_10_ and PM_2.5_ was found for *Alternaria*: effect estimates appeared close to null for PM levels below the median and significantly negative for PM levels above the median. An interaction with PM_10_ was also observed for grasses, estimates being significantly positive for concentrations above the median (11.1%, 95% CI: 4.2, 18.6), but not for levels below the median (2.4%, 95% CI: -4.1, 9.4). Ozone seemed, for its part, to modify the associations for hazel, hornbeam, birch and mugwort. For hazel and mugwort, ozone levels above the 85th percentile showed (non-significant) negative estimates, whereas estimates were closer to zero (and positive) for ozone levels below the 85th percentile. For hornbeam, birch and grasses, significant risks were only observed for ozone concentrations above the median (hornbeam: 0.8%, birch: 3.3%, grasses: 6.1%) or for ozone levels above the 85th percentile (hornbeam: 1.1%, birch: 7.0%, grasses: 7.9%,), although effect modification by this pollutant was only significant for hornbeam (ozone categorization according to the median) and for birch (ozone categorization according to the 85th percentile). Lastly, effect modification by NO_2_ was found for oak pollen, with a significant negative relationship for levels below the median (− 8.8%, 95% CI: −15.7, −1.5).

**Table 5:** Cumulative (lag 0–6) percentage change in asthma hospitalizations (95% confidence interval) associated with an interquartile range increase in pollen or fungal spore concentration, by air pollutant concentrations (below or above the 50th and 85th percentile), Brussels-Capital Region, 2008–2013

Percentage change (95% CI)												
Taxon	PM_2.5_ ≤ P50	PM_2.5_ > P50	P interaction	PM_2.5_ ≤ P85	PM_2.5_ > P85	P interaction	PM_10_ ≤ P50	PM_10_ > P50	P interaction	PM_10_ ≤ P85	PM_10_ > P85	P interaction
Alder	0.6 (−5.8, 7.4)	0.0 (−2.6, 2.6)	0.92	0.6 (−1.9, 3.2)	−2.2 (−6.7, 2.5)	0.76	0.3 (−6.2, 7.3)	−0.1 (−2.7, 2.6)	0.92	0.7 (−2.0, 3.5)	−2.9 (−7.0, 1.5)	0.54
Hazel	−0.9 (−5.7, 4.2)	0.9 (−2.2, 4.1)	0.68	0.2 (−2.8, 3.4)	0.4 (−4.9, 6.0)	0.21	−0.2 (−5.6, 5.4)	0.6 (−2.5, 3.9)	0.35	0.4 (−2.7, 3.6)	−0.4 (−5.1, 4.5)	0.37
Yew Cupressaceae	0.6 (−8.3, 10.4)	−2.6 (−7.3, 2.5)	0.62	−1.8 (−6.3, 3.0)	−9.3 (−19.0, 1.5)	0.63	0.7 (−8.4, 10.7)	−2.3 (−7.0, 2.7)	0.57	−2.7 (−7.8, 2.7)	−4.2 (−12.3, 4.5)	0.91
Ash	0.1 (−0.6, 0.8)	−0.6 (−1.4, 0.3)	0.51	−0.1 (−0.6, 0.5)	−0.6 (−4.1, 3.1)	0.89	0.0 (−0.9, 0.8)	−0.2 (−0.9, 0.4)	0.78	−0.1 (−0.7, 0.4)	−0.2 (−2.3, 2.0)	0.54
Hornbeam	0.9 (−0.1, 1.8)	0.7 (0.1, 1.3)*	0.53	0.9 (0.2, 1.6)*	0.1 (−2.0, 2.4)	0.38	0.7 (−0.2, 1.7)	0.7 (0.1, 1.4)*	0.63	0.9 (0.3, 1.6)*	1.1 (−0.9, 3.1)	0.83
Birch	3.6 (0.5, 6.8)*	2.8 (0.6, 5.2)*	0.82	3.3 (1.1, 5.6)*	4.6 (0.5, 8.9)*	0.51	3.6 (−0.1, 7.4)	2.8 (0.7, 5.1)*	0.2	3.3 (0.9, 5.7)*	2.9 (0.2, 5.7)*	0.94
Oak	−7.3 (−15.9, 2.2)	−4.2 (−10.4, 2.4)	0.51	−5.9 (−12.1, 0.6)	−3.7 (−11.3, 4.6)	0.59	−9.1 (−18.1, 0.9)	−3.3 (−10.0, 3.9)	0.61	−6.4 (−12.6, 0.3)	−2.5 (−9.9, 5.4)	0.74
Plantain	5.1 (−16.2, 31.8)	3.1 (−15.2, 25.2)	0.92	5.6 (−11.1, 25.5)	−0.5 (−37.1, 57.5)	0.91	9.0 (−14.7, 39.3)	2.3 (−15.0, 23.3)	0.49	5.5 (−11.2, 25.4)	−14.3 (−43.3, 29.5)	0.85
Grass	3.1 (−3.3, 10)	8.5 (1.7, 15.8)*	0.38	5.2 (−0.7, 11.4)	7.3 (−6.4, 23.0)	0.47	2.4 (−4.1, 9.4)	11.1 (4.2, 18.6)*	0.05°	5.6 (−0.3, 11.8)	7.2 (−6.5, 22.7)	0.69
Mugwort	2.2 (−3.7, 8.6)	−2.1 (−12.4, 9.5)	0.78	1.3 (−4.5, 7.5)	−38.9 (−68.8, 19.7)	0.15	2.0 (−3.9, 8.3)	−4.6 (−15.1, 7.3)	0.62	1.6 (−4.2, 7.7)	−33.2 (−69.3, 45.4)	0.24
*Alternaria*	0.0 (−3.1, 3.1)	− 4.7 (−9.1, 0.0)*	0.09°	−0.7 (−3.7, 2.4)	− 11.5 (−24.9, 4.2)	0.60	0.5 (−2.6, 3.7)	−6.1 (−10.6, −1.4)*	0.02°	−0.6 (−3.6, 2.5)	−8.0 (−20.8, 6.9)	0.25
*Cladosporium*	1.9 (−6.3, 10.8)	−0.5 (−11.1, 11.2)	0.64	1.3 (−6.8, 10.1)	−3.2 (−23.8, 22.9)	0.68	1.7 (−6.5, 10.6)	−5.3 (−15.6, 6.4)	0.61	1.6 (−6.5, 10.5)	−4.9 (−22.3, 16.4)	0.50
Percentage change (95% CI)												
Taxon	O_3_ ≤ P50	O_3_ > P50	P interaction	O_3_ ≤ P85	O_3_ > P85	P interaction	NO_2_ ≤ P50	NO_2_ > P50	P interaction	NO_2_ ≤ P85	NO_2_ > P85	P interaction
Alder	−0.6 (−3.5, 2.4)	1.1 (−2.0, 4.2)	0.81	0.3 (−2.2, 2.8)	−0.8 (−12.6, 12.5)	0.98	0.0 (−4.2, 4.3)	−0.2 (−3.0, 2.7)	0.95	0.7 (−1.9, 3.5)	−0.7 (−4.6, 3.4)	0.87
Hazel	0.5 (−3.0, 4.0)	−3.2 (−8.1, 2.0)	0.29	0.9 (−2.1, 4.0)	−11.5 (−31.0, 13.6)	0.04°	−2.8 (−7.3, 2.0)	0.8 (−2.5, 4.2)	0.19	0.5 (−2.7, 3.7)	0.6 (−3.6, 4.9)	1.00
Yew Cupressaceae	−6.1 (−12.3, 0.5)	−0.3 (−5.3, 4.9)	0.22	−2.2 (−6.7, 2.5)	−7.4 (−21.9, 9.8)	0.56	−1.3 (−7.5, 5.4)	−2.4 (−7.5, 3.0)	0.37	−1.0 (−5.9, 4.2)	−6.3 (−14.2, 2.2)	0.66
Ash	−1.0 (−2.5, 0.4)	0.1 (−0.5, 0.7)	0.22	−0.3 (−1.0, 0.4)	1.3 (−0.2, 2.8)	0.32	0.1 (−0.5, 0.7)	−0.4 (−1.2, 0.4)	0.83	0.0 (−0.5, 0.6)	−2.7 (−5.4, 0.1)	0.33
Hornbeam	1.6 (−0.3, 3.6)	0.8 (0.2, 1.3)*	0.12°	0.3 (−0.5, 1.0)	1.1 (0.3, 1.8)*	0.33	0.5 (−0.6, 1.7)	0.2 (−0.9, 1.4)	0.51	0.6 (0.1, 1.2)*	−6.5 (−14.7, 2.5)	0.25
Birch	2.7 (−2.5, 8.2)	3.3 (1.1, 5.5)*	0.40	1.9 (− 0.3, 4.2)	7.0 (3.5, 10.7)*	0.05°	3.6 (0.8, 6.5)*	3.0 (0.7, 5.4)*	0.65	3.0 (0.9, 5.2)*	−1.5 (−7.7, 5.2)	0.22
Oak	7.8 (−4.6, 21.8)	−6.2 (−12.1, 0.1)	0.16	−4.5 (−12.2, 3.7)	−5.8 (−12.0, 0.9)	0.96	−8.8 (−15.7, −1.5)*	−2.6 (−8.9, 4.2)	0.06°	−5.5 (−11.3, 0.8)	2.8 (−10.3, 17.9)	0.40
Plantain	−3.7 (−26.5, 26.3)	6.3 (−11.6, 27.8)	0.85	7.2 (−10.5, 28.3)	−4.0 (−26.2, 24.9)	0.84	8.9 (−10.4, 32.3)	−0.5 (−19.6, 23.0)	0.48	3.0 (−13.3, 22.3)	18.3 (−27.9, 94.0)	0.96
Grass	7.2 (−2.9, 18.3)	6.1 (0.2, 12.3)*	0.59	4.8 (−1.6, 11.7)	7.9 (1.1, 15.2)*	0.51	6.0 (0.0, 12.3)*	2.8 (−5.4, 11.7)	0.86	5.4 (−0.4, 11.7)	−8.0 (−53.3, 81.3)	0.4
Mugwort	−3.8 (−13.5, 7.0)	1.6 (−4.4, 7.9)	0.35	2.8 (−3.4, 9.4)	−7.4 (−16.6, 2.9)	0.14°	1.3 (−4.6, 7.6)	0.2 (−10.2, 11.9)	0.68	1.3 (−4.5, 7.5)	−16.5 (−59.0, 70.2)	0.95
*Alternaria*	−1.2 (−4.8, 2.5)	−0.8 (−4.0, 2.5)	0.40	−0.1 (−3.2, 3.1)	−3.3 (−8.0, 1.5)	0.63	−0.5 (−3.6, 2.7)	−2.6 (−7.1, 2.1)	0.41	−0.8 (−3.8, 2.3)	−6.8 (−20.4, 9.1)	0.57
*Cladosporium*	0.1 (−9.6, 10.9)	1.5 (−6.9, 10.7)	0.63	1.6 (−6.8, 10.7)	0.3 (−9.7, 11.4)	1.00	1.8 (−6.3, 10.7)	−4.3 (−14.8, 7.5)	0.51	1.3 (−6.8, 10.1)	8.6 (−16.7, 41.6)	0.26

Note: Models adjusted for seasonal and long-term trends, day of the week, public holidays and a DLNM cross-basis for mean temperature and relative humidity

* *P* < 0.05

° *P* interaction < 0.15 (significant effect modification by air pollutant)

The use of unconstrained distributed lag models gave similar results (see Additional file 1). A more stringent adjustment for temporal trends (knots every 15 days instead of every 30 days) produced slightly larger confidence intervals for hornbeam (0.7%, 95% CI: -0.1, 1.5) and grasses (7.0%, 95% CI: -0.8, 15.5) whereas a less stringent adjustment (knots every 60 days) resulted in a small decrease in estimates for birch (2.4%, 95% CI: 0.5, 4.4) and hornbeam (0.5%, 95% CI: 0.0, 1.0) but in an increase in the estimate for grasses (9.7%, 95% CI: 5.3, 14.2). The less stringent adjustment for temporal trends also produced significant negative estimates for *Alternaria* (-4.6%, 95% CI: -6.8, -2.4) and *Cladosporium* (-11.7%, 95% CI: -16.7, -6.3).

## Discussion

This time series analysis investigated the relationships between daily asthma hospitalizations and daily outdoor aeroallergen levels in the Brussels-Capital Region for the 2008–2013 period. 11 pollen and two fungal spore taxa relevant for the study area were tested.

After adjustment for meteorological factors, seasonality, long-term trends, day of the week and public holidays, grass, birch and hornbeam pollen concentrations were positively and significantly associated with asthma hospitalizations. These associations did not appear to be confounded by air pollution, general respiratory infections or influenza epidemics. Grasses showed overall the strongest association with an increase of 5.9% (95% CI: 0.0, 12.0) in asthma admissions for an interquartile range increase in pollen concentration. This relationship has also been observed in the United Kingdom [16, 29], Spain [12], the United States [15], Australia [11] and Canada [10, 14]. In London, the change in asthma admissions for a 0-95th percentile increase in pollen exposure reached 17.23% (95% CI: 8.93, 25.54) at 4-day lag [16]. These results are consistent with the great allergy potency and ubiquity of grass pollen, recognized as the main causative agent of pollinosis in Europe [30]. In Belgium (Ghent), 25.5% of patients suffering from allergic reactions to inhalant allergens were sensitized to grass pollen according to a Global Asthma and Allergy European Network (GA2LEN) study [31] (a figure confirmed by another study [32]; European average: 37.8% [31]). This allergen was responsible for the second highest rate of sensitization in the country, just behind house dust mites [31]. This sensitization might be, however, species-specific and some researchers encourage to go beyond the current measurement method of the “total” grass pollen [16].

A significant increase in asthma hospitalizations with increasing pollen concentrations was also observed for birch (3.2%, 95% CI: 1.1, 5.3). This result agrees with findings from the United States (increase close to 35%, for a 0-98th percentile increase in pollen levels) [13] and the United Kingdom (increase equal to 0.78%, 95% CI: 0.15, 1.42, for a 10 unit increase in pollen concentration) [33]. Birch is considered as the main tree taxon responsible for the pollinosis risk in Belgium [31]. It is widely spread over the territory in the form of silver birch (*Betula pendula* syn.: *B. verrucosa* and *B. alba*) or downy birch (*B. pubescens*). It constitutes the principal source of tree pollen grains [34], whose concentrations tended to increase the last 35 years [20, 21].

Asthma hospitalizations were significantly associated with hornbeam pollen concentrations too (0.7%, 95% CI: 0.2, 1.3). To the best of our knowledge, only one similar study investigated the individual contribution of this taxon [35]. This work, carried out in Croatia, demonstrated a strong relationship between asthma admissions and hornbeam pollen concentrations (increase of 21%, 95% CI: 11, 30, for a 95-99th percentile increase in hornbeam pollen levels). Despite that Swiss clinical researches identified asthmatic patients to be specifically sensitized to hornbeam allergens [36], associations for hornbeam may be confounded by birch due to the overlap in their pollen season and potential cross-allergenicity (hornbeam and birch belonging to the same family).

Contrary to previous similar works [10, 12, 13, 15, 17, 29, 37, 38] and despite their recognized allergy potency, no significant positive relationships were observed for alder, hazel, ash, oak, plantain or the fungal spore taxa *Alternaria* and *Cladosporium*.

Overall, conclusions appear consistent with a previous study which investigated the short-term associations between allergy medication sales and outdoor aeroallergen concentrations in the Brussels-Capital Region [39]. These analyses highlighted strong positive relationships for grass and birch taxa and, to a lesser extent, for hornbeam, ash and oak (rather consistent negative associations were observed for *Alternaria* and *Cladosporium* too).

Significant effect modification by age was observed for plantain, grasses, mugwort, *Alternaria* and *Cladosporium.* For all of these taxa except plantain, a trend of lower effect estimates in the group 60 or more years compared to younger age groups was demonstrated. Two previous works from North America showed for various tree species the strongest associations for the age group 5–17 years [13, 15]. Also, the previously mentioned study in the Brussels region highlighted stronger relationships between allergy medication sales and aeroallergen concentrations for young and intermediate age groups [39]. These findings might be explained by differences in disease management: the younger patients are more likely to be undiagnosed and in this way may be more vulnerable to aeroallergen peaks than the older ones. In addition, elderlies may be less exposed to outdoor aeroallergens due to mobility limitations. The risk of misdiagnosis leading to misclassification issues should also be considered. It may be higher among very young and old patients, more at risk for acute respiratory infections or chronic obstructive pulmonary disease (COPD) (knowing exclusion of individuals less than five-year-old did not significantly affect the results in this study). More generally, other sociodemographic factors including gender and education might play a key role. A Canadian study specifically showed stronger risk of asthma hospitalizations related to aeroallergen levels for younger males than older ones and an opposite trend for females [40]. Also, associations were only significant for the lowest educated patients. Unfortunately, the impact of these factors could not be investigated in the present study because of a lack of information and/or a re-identification risk.

Consistent with other studies, no confounding effect of air pollution was demonstrated here [10–12, 15, 17]. Signs of effect modification by air pollutants were, however, notable: high atmospheric PM_10_ and ozone concentrations seemed to potentiate the risk of hospitalizations associated with the grass and birch taxa respectively. These interactions have been observed in other population studies [10, 41, 42] but also experimentally [43]. In Canada, risks of asthma admissions associated with the presence of airborne tree pollen and various fungal spores were consistently higher on days with high concentrations of PM compared to days with low levels (observed interactions for other pollutants were less consistent) [41]. The biological plausibility of these interactions is supported by various potential underlying mechanisms. First of all, air pollutants have been identified as responsible for airway tissue injury and impaired mucociliary clearance, facilitating the contact between the aeroallergens and the immune system [44]. This comes with inflammation, promoting the release of various mediators involved in asthma expression. Besides, several studies have highlighted the ability of air pollutants (especially carbon dioxide) to increase plant biomass and pollen amounts [45–47]. These compounds may in addition modulate the allergenicity of some airborne allergens through attachment (modifying their process by the immune system). Lastly, some of them may act as carriers and/or induce the rupture of pollen grains, generating smaller allergenic particles able to penetrate the respiratory tract more deeply [43, 44, 47]. These “adjuvant effects” contribute to a lowering of the concentration threshold associated with respiratory symptoms, leading to higher risk of allergic sensitization among healthy individuals and symptom exacerbations in already allergic subjects. Such synergistic effects have, however, not been demonstrated here for all the studied aeroallergens and are not systematically observed in other research works [15, 29, 33, 35]. This might be explained by a lack of statistical power associated with sometimes low variations in air pollutant concentrations during the often short peak pollen period.

We also considered potential confounding by influenza epidemics and respiratory viral infections. The latter are recognized as a major cause of asthma symptoms aggravation around September. It is probably behind 50–80% of exacerbation episodes, with an apparent higher risk for individuals suffering from allergic asthma compared with individuals having a non-allergic form of the disease [48–51]. In Canada, respiratory infections explained 14% of the variance in asthma hospitalizations registered for preschool children [51]. The influence of this factor on the present results is unlikely: analyses filtered out seasonal trends and inclusion of variables representative of influenza epidemics or of hospital admissions due to respiratory infections did not noticeably change the results. Besides, the significant increase in asthma admissions associated with grass pollen levels persisted after restriction of the analyses to months April–August (8.5%, 95% CI: 0.5, 17.1). Possible role of a decline in population size during summer should, for its part, be captured with the correction for seasonality.

More generally, even in case of similar aeroallergen concentrations, allergy and symptom exacerbation risks can vary due to a wide range of factors: other aeroallergens simultaneously present, air pollution levels, weather conditions, season or difference with regard to population sensitization or susceptibility patterns. In this framework, strong geographical variations in skin test results have been observed worldwide. Differences are notably high for *Alternaria* and *Cladosporium* whose prevalence of sensitization among the general population does not exceed 2.5% in Belgium (Antwerp) [52] while it may vary between 4.8% and 12.7% in Canada [53] (where associations between Ascomycetes spore levels and asthma admissions were observed [10, 18]). Besides, methodological disparities have to be taken into account when comparing studies: differences in the selected study period and population (e.g. with respect to age), pollen monitoring methods (not standardized) [54], confounding variables considered, statistical model, selected lags, shape of the exposure-response function, etc. Finally, results might be affected by variations in local healthcare strategy (disease diagnosis, prevention or management) or cultural habits with regard to medication use and hospital services. This highlights how conclusions are context-specific and extrapolation to other geographical areas sensitive.

Few time series studies have investigated the short-term relationships between ambient aeroallergen concentrations and asthma hospitalizations in the ecological context of Northern Europe. To our knowledge, all of them focused on the United Kingdom [16, 29, 33, 38]. This kind of study was conducted here for the first time in Belgium which is characterized by high hospitalization rates for asthma [3, 5]. It distinguishes itself by the diversity of aeroallergens considered. In particular, contrary to some previous works [10, 41, 42], analyses were carried out for individual taxa instead of total tree pollen or fungal spore groups, allowing a finer assessment of health risks. Besides, various potential confounders and effect modifiers were examined. The present results were robust to numerous sensitivity analyses and strengthen conclusions derived from a similar work focusing on allergy symptoms [39].

Some limitations should however be highlighted. First, because of the ecological and observational nature of this study, results should be interpreted at the population level and cannot be read as causal associations. Unmeasured confounders may still influence the estimates even if time-series design controls for important individual risk factors that do not vary with time (such as tobacco consumption, genetic predispositions, etc.). Second and following on from this, one should highlight approximations made regarding exposure measurement. Indeed, aeroallergens data were derived from a unique spore sampler, located on the top of a building and potentially influenced by local environmental characteristics. Analyses assumed an even exposure to these substances over the study area, ruling out the impact of time spent outdoor by each person, spatial heterogeneity in sources, physical barrier to transport and leading to potential exposure misclassification. Some studies have, nevertheless, concluded that despite rather poor representativeness of personal exposure, concentrations derived from stationary pollen traps correlate well with patients’ symptomology [55]. In general, these traps might be representative of 30–40 km regions [56, 57]. The same limitation applies for air pollutants as one single population-weighted average concentration was used for the whole study area. Nonetheless, restriction of the overall analyses to smaller areas in a previous similar study did not substantially modify the results (investigation not possible here due to data aggregation) [39]. Third, as in other studies of this kind and already mentioned, a risk of disease misclassification exists due to the rather non-specificity/heterogeneity of asthma symptoms. This risk may go both ways with the exclusion of true asthma cases and inclusion of false cases. It might be higher in autumn or winter (when respiratory infections increase) and explain the absence of association observed for alder and hazel despite the allergenic properties of these taxa and the sensitization of Belgian patients [30, 31]. This risk may also be higher for very young patients (for whom asthma diagnosis is uncertain and more subject to respiratory infections) and old patients (for whom asthma symptoms may be confused with COPD ones). More generally, limiting the analyses to asthma admissions lead to focus on patients suffering from the most severe forms of the disease or on weakened individuals.

Despite these limitations, the public health impact of outdoor aeroallergens should not be underestimated. Asthma is indeed one of the leading causes of morbidity worldwide and allergens exposure is almost unavoidable [58]. Considering the high admission rates registered for this pathology in Belgium [3, 5], actions should be undertaken. In this framework, it would have been interesting to have information on individual’s consumption of long-term control and quick-relief medicines in order to assess the percentage of persons hospitalized due to non-diagnosis or due to loss of disease control. Data on patients’ sensitization would have been of course valuable too. Such information would support targeted actions, at different levels. This could include: better disease diagnosis, improved patients’ compliance with their treatment, development of accurate aeroallergen level forecast and increased/more targeted warning communication (daily information on pollen and fungal spore concentrations is currently relayed through specialized websites, newsletters and mobile app in Belgium) [22]. These actions should not dismiss the concomitant impact of air pollution and individual sensitivity.

## Conclusions

This study suggests that increasing concentrations of airborne grass, birch and hornbeam pollen can severely exacerbate asthma symptoms, leading to hospitalizations in the Brussels-Capital Region. Despite some inconsistencies, a trend of stronger associations between aeroallergens and asthma hospitalizations in individuals younger than 60 years and on days with high air pollution levels was observed. These results highlight the importance of a regular monitoring for outdoor aeroallergen levels as well as the need for additional efforts to anticipate and reduce the health risks associated with these compounds.

## Additional file

Additional file 1: **Table S1.** Spearman’s correlation coefficients among aeroallergens, Brussels-Capital Region, 2008–2013. **Table S2.** Confounding by air pollutants, influenza epidemics and general respiratory infections - Cumulative (lag 0–6 days) percentage change (95% confidence interval) in asthma hospitalizations associated with an interquartile range increase in pollen or fungal spore concentrations, Brussels-Capital Region, 2008–2013. **Table S3.** Sensitivity analyses - Cumulative (lag 0–6 days) percentage change (95% confidence interval) in asthma hospitalizations associated with an interquartile range increase in pollen or fungal spore concentrations, Brussels-Capital Region, 2008–2013. **Figure S1.** Cumulative (lag 0–6) exposure-response (ER) functions for the association between asthma admissions and some aeroallergen concentrations - Functions modelled using a natural cubic spline–natural cubic spline DLNM with 3 degrees of freedom (df) for the ER function and 4 df for the lag structure. Relative risks (RR) are relative to the reference value of 0 grains/m^3^. The vertical dotted lines represent the 75th, 95th and 99th percentiles of the pollen concentrations). (DOCX 37 kb)

## Abbreviations

CI: Confidence Interval
COPD: Chronic Obstructive Pulmonary Disease
df: Degrees of Freedom
DL(N)M: Distributed Lag (Non-linear) Model
