# Supplementary material for: Relationships between aeroallergen levels and hospital admissions for asthma in the Brussels-Capital Region: a daily time series analysis
Source: Environ Health. 2018 Apr 11;17:35. doi: 10.1186/s12940-018-0378-x (PMC5896062; doi:10.1186/s12940-018-0378-x)
Supplement: Supplementary file 1 — Table S1. Spearman’s correlation coefficients among aeroallergens, Brussels-Capital Region, 2008–2013. Table S2. Confounding by air pollutants, influenza epidemics and general respiratory infections - Cumulative (lag 0–6 days) percentage change (95% confidence interval) in asthma hospitalizations associated with an interquartile range increase in pollen or fungal spore concentrations, Brussels-Capital Region, 2008–2013. Table S3. Sensitivity analyses - Cumulative (lag 0–6 days) percentage change (95% confidence interval) in asthma hospitalizations associated with an interquartile range increase in pollen or fungal spore concentrations, Brussels-Capital Region, 2008–2013. Figure S1. Cumulative (lag 0–6) exposure-response (ER) functions for the association between asthma admissions and some aeroallergen concentrations - Functions modelled using a natural cubic spline–natural cubic spline DLNM with 3 degrees of freedom (df) for the ER function and 4 df for the lag structure. Relative risks (RR) are relative to the reference value of 0 grains/m3. The vertical dotted lines represent the 75th, 95th and 99th percentiles of the pollen concentrations). (DOCX 37 kb) [file 12940_2018_378_MOESM1_ESM.docx]

**Supplementary material**

*Table S1: Spearman’s correlation coefficients among aeroallergens, Brussels-Capital Region, 2008–2013*

|  | Alder | Hazel | Yew Cupressaceae | Ash | Hornbeam | Birch | Oak | Plantain | Grass | Mugwort | *Alternaria* | *Cladosporium* |
| --- | --- | --- | --- | --- | --- | --- | --- | --- | --- | --- | --- | --- |
| Alder | 1 |  |  |  |  |  |  |  |  |  |  |  |
| Hazel | 0.77 | 1 |  |  |  |  |  |  |  |  |  |  |
| Yew Cupressaceae | 0.43 | 0.36 | 1 |  |  |  |  |  |  |  |  |  |
| Ash | 0.24 | 0.17 | 0.49 | 1 |  |  |  |  |  |  |  |  |
| Hornbeam | 0.11 | 0.06 | 0.36 | 0.65 | 1 |  |  |  |  |  |  |  |
| Birch | 0.01 | -0.05 | 0.37 | 0.63 | 0.63 | 1 |  |  |  |  |  |  |
| Oak | -0.06 | -0.10 | 0.20 | 0.34 | 0.35 | 0.66 | 1 |  |  |  |  |  |
| Plantain | -0.14 | -0.14 | 0.12 | -0.08 | -0.02 | 0.11 | 0.13 | 1 |  |  |  |  |
| Grass | -0.27 | -0.28 | 0.17 | -0.11 | -0.03 | 0.20 | 0.25 | 0.45 | 1 |  |  |  |
| Mugwort | -0.15 | -0.15 | 0.07 | -0.13 | -0.10 | -0.14 | -0.12 | 0.26 | 0.26 | 1 |  |  |
| *Alternaria* | -0.29 | -0.28 | 0.06 | -0.21 | -0.13 | -0.12 | -0.13 | 0.30 | 0.47 | 0.46 | 1 |  |
| *Cladosporium* | -0.32 | -0.32 | 0.05 | -0.23 | -0.13 | -0.08 | -0.04 | 0.34 | 0.59 | 0.41 | 0.78 | 1 |

*Table S2: Confounding by air pollutants, influenza epidemics and general respiratory infections*

*Cumulative (lag 0‒6 days) percentage change (95% confidence interval) in asthma hospitalizations associated with an interquartile range increase in pollen or fungal spore concentrations, Brussels-Capital Region, 2008-2013*

|  | **Percentage change (95% CI)** | | | | |
| --- | --- | --- | --- | --- | --- |
| **Taxon** | **Main model** | **+ PM_2.5_** | **+ PM_10_** | **+ O_3_** | **+ NO_2_** |
| Alder | 0.2 (-2.2, 2.7) | 0.3 (-2.1, 2.8) | 0.3 (-2.2, 2.8) | 0.1 (-2.4, 2.6) | 0.3 (-2.2, 2.8) |
| Hazel | 0.5 (-2.5, 3.6) | 0.7 (-2.3, 3.7) | 0.7 (-2.3, 3.7) | 0.4 (-2.6, 3.5) | 0.5 (-2.5, 3.5) |
| Yew Cupressaceae | -2.5 (-6.9, 2.1) | -2.5 (-6.9, 2.2) | -2.4 (-6.9, 2.2) | -2.3 (-6.7, 2.3) | -2.4 (-6.8, 2.3) |
| Ash | 0.0 (-0.6, 0.5) | -0.1 (-0.6, 0.5) | 0.0 (-0.6, 0.5) | -0.1 (-0.6, 0.5) | -0.1 (-0.6, 0.4) |
| Hornbeam | 0.7 (0.2, 1.3)* | 0.8 (0.2, 1.3)* | 0.8 (0.2, 1.4)* | 0.8 (0.2, 1.3)* | 0.8 (0.2, 1.4)* |
| Birch | 3.2 (1.1, 5.3)* | 3.2 (1.1, 5.3)* | 3.2 (1.1, 5.3)* | 3.3 (1.2, 5.5)* | 3.3 (1.2, 5.5)* |
| Oak | -5.6 (-11.3, 0.5) | -4.9 (-11.0, 1.6) | -5.3 (-11.3, 1.1) | -5.8 (-11.9, 0.6) | -5.5 (-11.4, 0.8) |
| Plantain | 4.1 (-11.9, 22.9) | 6.1 (-10.3, 25.5) | 5.7 (-10.7, 25) | 4.1 (-11.9, 23.1) | 9.1 (-8.0, 29.3) |
| Grass | 5.9 (0.0, 12.0)* | 6.6 (0.7, 12.8)* | 6.5 (0.7, 12.8)* | 6.0 (0.1, 12.2)* | 5.3 (-0.5, 11.5) |
| Mugwort | 1.4 (-4.4, 7.6) | 0.9 (-4.8, 7.0) | 1.0 (-4.7, 7.1) | 1.5 (-4.4, 7.7) | 1.2 (-4.5, 7.3) |
| *Alternaria* | -0.8 (-3.8, 2.3) | -0.6 (-3.6, 2.5) | -0.7 (-3.7, 2.4) | -0.8 (-3.8, 2.3) | -0.7 (-3.7, 2.4) |
| *Cladosporium* | 1.2 (-6.9, 10.0) | 1.9 (-6.3, 10.8) | 1.6 (-6.6, 10.4) | 1.2 (-6.9, 10.1) | 1.3 (-6.9, 10.1) |

|  | **Percentage change (95% CI)** | | |
| --- | --- | --- | --- |
| **Taxon** | **Main model** | **+ Influenza epidemics** | **+ General respiratory infections** |
| Alder | 0.2 (-2.2, 2.7) | 0.1 (-2.3, 2.6) | 0.2 (-2.2, 2.7) |
| Hazel | 0.5 (-2.5, 3.6) | 0.5 (-2.5, 3.6) | 0.5 (-2.5, 3.6) |
| Yew Cupressaceae | -2.5 (-6.9, 2.1) | -2.3 (-6.8, 2.3) | -2.5 (-6.9, 2.1) |
| Ash | 0.0 (-0.6, 0.5) | 0.0 (-0.6, 0.5) | 0.0 (-0.6, 0.5) |
| Hornbeam | 0.7 (0.2, 1.3)* | 0.7 (0.2, 1.3)* | 0.7 (0.2, 1.3)* |
| Birch | 3.2 (1.1, 5.3)* | 3.2 (1.2, 5.3)* | 3.2 (1.1, 5.3)* |
| Oak | -5.6 (-11.3, 0.5) | -5.6 (-11.3, 0.5) | -5.6 (-11.4, 0.5) |
| Plantain | 4.1 (-11.9, 22.9) | 4.2 (-11.8, 23.1) | 4.2 (-11.9, 23.1) |
| Grass | 5.9 (0.0, 12.0)* | 6.0 (0.1, 12.2)* | 5.9 (0.1, 12.1)* |
| Mugwort | 1.4 (-4.4, 7.6) | 1.6 (-4.2, 7.7) | 1.3 (-4.5, 7.5) |
| *Alternaria* | -0.8 (-3.8, 2.3) | -0.8 (-3.8, 2.3) | -0.8 (-3.8, 2.3) |
| *Cladosporium* | 1.2 (-6.9, 10.0) | 1.2 (-6.9, 10.0) | 1.2 (-6.9, 10.0) |

*Note: All models are adjusted for seasonal and long-term trends, day of the week, public holidays and DLNM cross-bases for mean temperature and relative humidity.*

*** *P < 0.05.*

*Table S3: Sensitivity analyses*

*Cumulative (lag 0‒6 days) percentage change (95% confidence interval) in asthma hospitalizations associated with an interquartile range increase in pollen or fungal spore concentrations, Brussels-Capital Region, 2008-2013*

|  | **Percentage change (95% CI)** | | | |
| --- | --- | --- | --- | --- |
| **Taxon** | **Main model** | **Unrestricted lag** | **Knot every 15 days** | **Knot every 60 days** |
| Alder | 0.2 (-2.2, 2.7) | 0.2 (-2.2, 2.8) | -1.6 (-4.5, 1.4) | 0.5 (-1.6, 2.6) |
| Hazel | 0.5 (-2.5, 3.6) | 0.5 (-2.5, 3.6) | -0.1 (-4.0, 4.0) | 1.0 (-1.7, 3.7) |
| Yew Cupressaceae | -2.5 (-6.9, 2.1) | -2.5 (-6.9, 2.1) | -3.1 (-7.8, 1.9) | -3.3 (-7.5, 1.2) |
| Ash | 0.0 (-0.6, 0.5) | 0.0 (-0.6, 0.5) | -0.4 (-1.1, 0.4) | -0.1 (-0.6, 0.4) |
| Hornbeam | 0.7 (0.2, 1.3)* | 0.7 (0.2, 1.3)* | 0.7 (-0.1, 1.5) | 0.5 (0.0, 1.0) |
| Birch | 3.2 (1.1, 5.3)* | 3.1 (1.0, 5.2)* | 3.4 (0.6, 6.2)* | 2.4 (0.5, 4.4)* |
| Oak | -5.6 (-11.3, 0.5) | -6.0 (-11.8, 0.1) | -1.1 (-8.2, 6.7) | -3.9 (-8.8, 1.2) |
| Plantain | 4.1 (-11.9, 22.9) | 3.6 (-12.4, 22.4) | 1.3 (-17.8, 24.8) | 1.2 (-13.5, 18.4) |
| Grass | 5.9 (0.0, 12.0)* | 5.7 (-0.1, 11.8) | 7.0 (-0.8, 15.5) | 9.7 (5.3, 14.2)* |
| Mugwort | 1.4 (-4.4, 7.6) | 1.3 (-4.5, 7.5) | 3.2 (-7.0, 14.5) | -0.3 (-4.5, 4.1) |
| *Alternaria* | -0.8 (-3.8, 2.3) | -0.8 (-3.7, 2.3) | -0.9 (-5.1, 3.4) | -4.6 (-6.8, -2.4)* |
| *Cladosporium* | 1.2 (-6.9, 10.0) | 1.1 (-7.0, 9.8) | -4.4 (-15.1, 7.6) | -11.7 (-16.7, -6.3)* |

|  | **Percentage change (95% CI)** | |
| --- | --- | --- |
| **Taxon** | **0-14 year patients** | **5-14 year patients** |
| Alder | -1.0 (-3.7, 1.7) | -0.9 (-3.8, 2.0) |
| Hazel | -0.1 (-3.3, 3.2) | 0.5 (-2.9, 4.1) |
| Yew Cupressaceae | -3.9 (-8.8, 1.4) | -5.3 (-10.6, 0.4) |
| Ash | -0.1 (-0.7, 0.5) | -0.4 (-1.1, 0.3) |
| Hornbeam | 0.7 (0.1, 1.3)* | 0.8 (0.2, 1.4)* |
| Birch | 3.3 (1.2, 5.5)* | 3.0 (0.7, 5.4)* |
| Oak | -5.7 (-11.6, 0.5) | -5.2 (-11.7, 1.7) |
| Plantain | 7.4 (-9.9, 28.1) | -4.1 (-21.4, 17.1) |
| Grass | 5.2 (-0.9, 11.5) | 5.9 (-0.8, 13.0) |
| Mugwort | 1.3 (-4.8, 7.9) | 1.1 (-5.5, 8.1) |
| *Alternaria* | -0.3 (-3.4, 2.9) | -0.3 (-3.5, 3.1) |
| *Cladosporium* | 2.3 (-6.1, 11.3) | -0.3 (-8.9, 9.0) |

*Note: All models are adjusted for seasonal and long-term trends, day of the week, public holidays and DLNM cross-bases for mean temperature and relative humidity.*

*** *P < 0.05.*


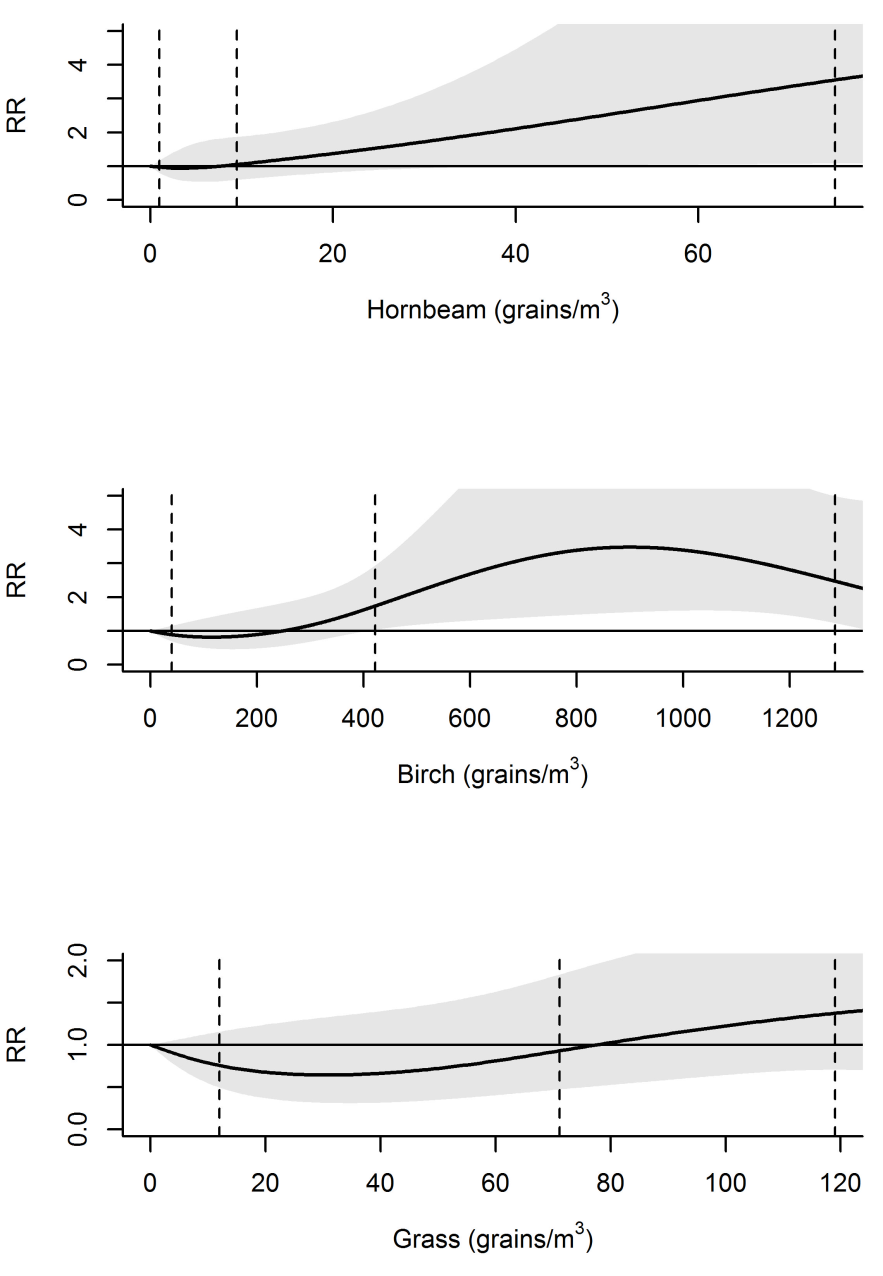


*Figure S1: Cumulative (lag 0–6) exposure-response (ER) functions for the association between asthma admissions and some aeroallergen concentrations*

*Functions modelled using a natural cubic spline–natural cubic spline DLNM with 3 degrees of freedom (df) for the ER function and 4 df for the lag structure. Relative risks (RR) are relative to the reference value of 0 grains/m^3^. The vertical dotted lines represent the 75^th^, 95^th^ and 99^th^ percentiles of the pollen concentrations)*
